# Spinal Cystic Echinococcosis – A Systematic Analysis and Review of the Literature: Part 1. Epidemiology and Anatomy

**DOI:** 10.1371/journal.pntd.0002450

**Published:** 2013-09-19

**Authors:** Andreas Neumayr, Francesca Tamarozzi, Sam Goblirsch, Johannes Blum, Enrico Brunetti

**Affiliations:** 1 Swiss Tropical and Public Health Institute, Basel, Switzerland; 2 Division of Infectious and Tropical Diseases, University of Pavia, IRCCS S. Matteo Hospital Foundation, World Health Organization Collaborating Centre for Clinical Management of Cystic Echinococcosis, Pavia, Italy; 3 Department of Medicine and Pediatrics, University of Minnesota, Minneapolis, Minnesota, United States of America; Universidad Nacional Autónoma de México, Mexico

## Abstract

Bone involvement in human cystic echinococcosis (CE) is rare, but affects the spine in approximately 50% of cases. Despite significant advances in diagnostic imaging techniques as well as surgical and medical treatment of spinal CE, our basic understanding of the parasite's predilection for the spine remains incomplete. To fill this gap, we systematically reviewed the published literature of the last five decades to summarize and analyze the currently existing data on epidemiological and anatomical aspects of spinal CE.

## Author Summary

Spinal cystic echinococcosis (CE) is a rare but malignant form of a truly neglected tropical disease. Despite significant advances in diagnostic imaging techniques as well as surgical and medical treatment of spinal CE, our basic understanding of the parasite's predilection for the spine remains poor at best. Information on the influence of parasite and host specific factors on anatomical manifestations and evolution of CE is currently lacking. We systematically reviewed all published case reports and case series of spinal CE from 1965 until 2012 to summarize and analyze the epidemiological and anatomical aspects of the disease and discuss the findings in light of the existing data.

## Introduction

Hydatid disease or cystic echinococcosis (CE), caused by the larval stage of the cestode *Echinococcus granulosus*, is a cosmopolitan parasitic zoonosis occurring on every continent except Antarctica. Hydatid (Greek for ‘watery cyst’) disease was already recognized by Hippocrates over 2000 years ago and in 1807 Churrier made the first description of spinal hydatidosis, roughly 100 years after Bidloo (1708) discovered the existence of a bony form of the disease [1].

The parasite's lifecycle involves two hosts. The definitive host is usually the dog (but may be another carnivore), where the adult parasite lives - attached by hooklets and suckers to the mucosa - in the proximal small bowel. The eggs of the parasite are shed with the host's feces into the environment where the intermediate host, usually a sheep (but may be some other herbivore), gets infected when grazing on contaminated ground. After ingestion of the egg, the embryo (*oncosphere*) hatches, penetrates the intestinal mucosa, enters into the host's circulatory system (via venous and lymphatic pathways), and (if not destroyed by the host's immune response) develops into the characteristic vesicular *metacestode* when reaching a suitable anatomical site. This stage of the parasite is typically a unilocular, fluid-filled cystic lesion (‘hydatid’, ‘hydatid cyst’), which grows expansively by concentric enlargement (increasing in diameter from 1–5 cm per year) within the affected organ and harbors the infective *protoscolices*. When the definitive host feeds on infected viscera, the cycle is complete [2].

In the accidental human intermediate host, the characteristic cystic lesions are mainly found in the liver (∼70%) and the lungs (∼20%), but virtually any part of the body may be affected, including the bone (∼0.5–4%). The central nervous system (which is involved in ∼3% of all cases) and the vertebral column (which is involved in ≥50% of the ∼0.5–4% of cases affecting the bone) [3]–[6] are particularly vulnerable given the sequelae that result from their involvement. ‘Spinal CE’ (involvement of the spinal cord, the spine, or both structures) is associated with a high degree of morbidity, disability, and mortality and the prognosis has often been compared to that of malignancies (‘*le cancer blanc*’ [7]).

We systematically reviewed all published case reports and case series of spinal CE from 1965 until 2012 to summarize and analyze the epidemiological and anatomical aspects of the disease and discuss the findings in light of the existing data.

## Methods

**Figure 1:**
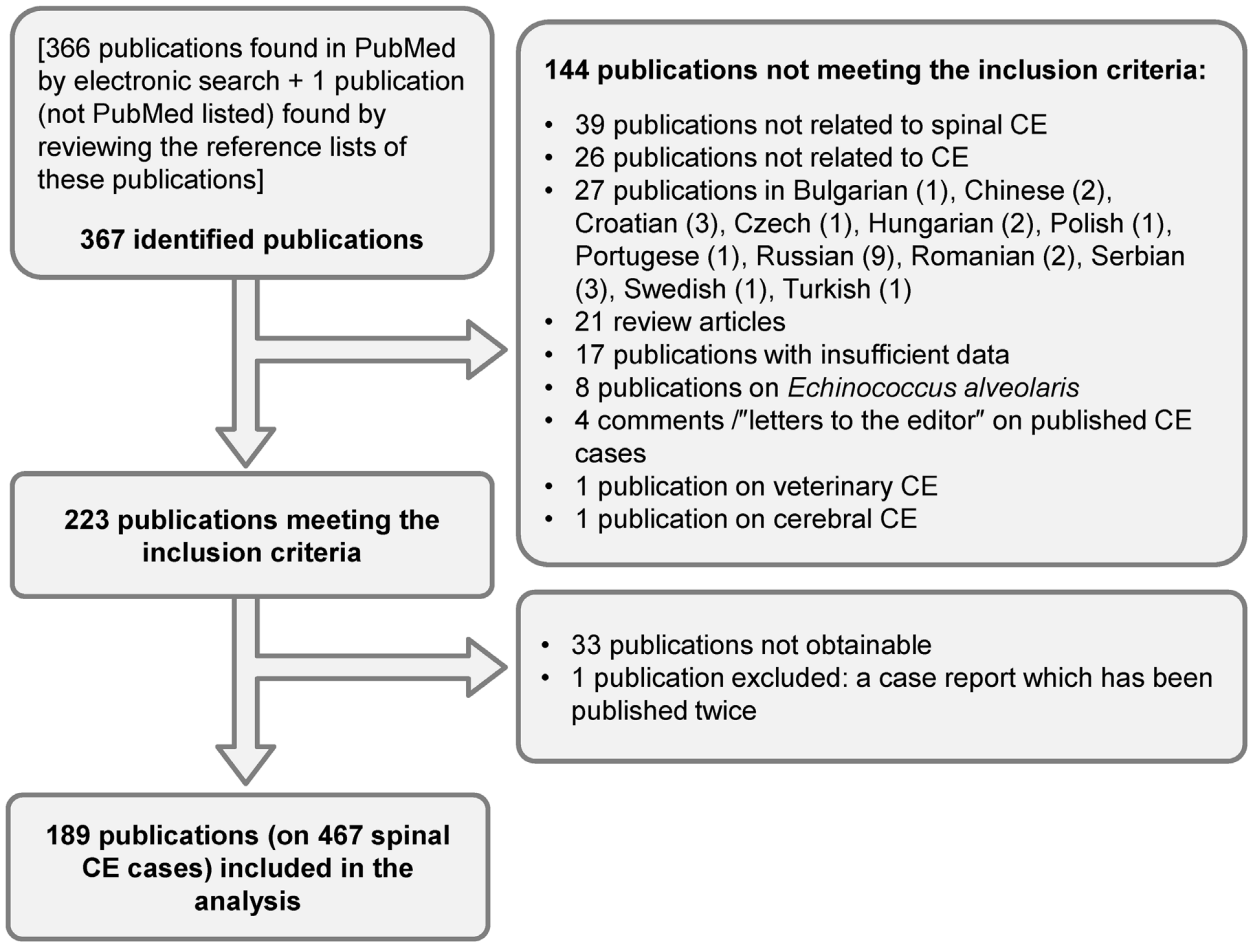
Flow diagram of search and selection of eligible publications.

We performed a PubMed (MEDLINE) search of the literature using the key words ‘spinal echinococcosis’, ‘spinal hydatidosis’, ‘spinal hydatid disease’, ‘spinal echinococcal cyst’, ‘spinal cystic echinococcosis’ and reviewed the obtained references published from 1965 until July 1st 2012 (figure 1; references S1). The year 1965 was chosen, as it proved difficult to obtain articles before this year.

All publications on clinical cases and case series of human spinal echinococcosis published in English, French, German, Italian, and Spanish were collected. When the original article was not obtainable but the abstract contained data on anatomy, treatment approach or therapeutic outcome, the publication was included in the analysis. In addition, the reference lists of the collected publications were screened for supplementary (not PubMed listed) case reports on spinal CE eligible for analysis. The collected data included patient's age, sex, if applicable manifestations, interventions and time frame of previous spinal or extraspinal CE, cyst number, cyst location(s), and involved anatomical structures.

The extracted data was entered into Microsoft Excel-files (Version 2002) and later transformed into SPSS-files (Version 16.0.0, 2007) for analysis. Data on the age of the patients, follow-up periods and recurrence periods was summarized as medians and ranges and, if applicable, analysed by using the Mann-Whitney U test. Nominal data was summarized as frequencies and percentages and analysed by χ^2^-test. A p-value <0.05 was considered statistically significant.

## Results

Of the 367 publications identified by electronic search, 189 publications (on 467 cases of spinal CE) were included in the analysis (figure 1).

**Figure 2:**
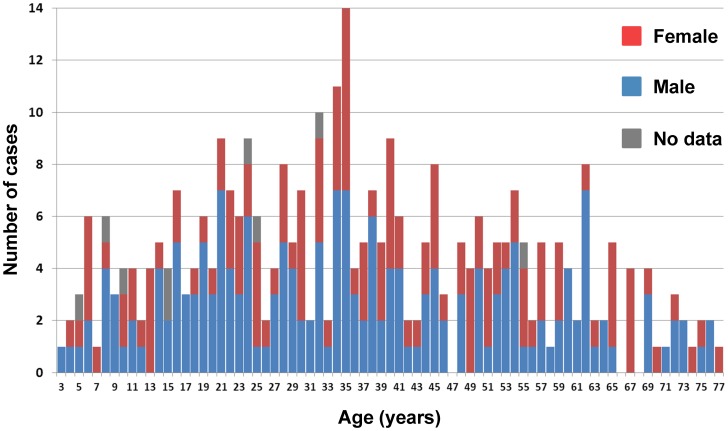
Age distribution of spinal CE (data on 325 cases).

Individual data on the patient's age was available for 325 cases, on the gender for 408 cases (232 male, 176 females) and on age and gender for 316 (186 male, 130 female) cases. The discrepancy between data on age and on gender is due to case series, where data on gender was available but data on age was limited to the mean or median of the case series. The overall median age was 35 years (range 3–77 years) without significant difference between male (median 35 years; range 3–76 years) and female cases (median 36 years; range 4–77 years)(figure 2).

Data on the number of cysts was available for 243 of the 467 spinal CE cases: 56 (23%) presented with a single cyst, 187 (77%) presented with multiple cysts.

**Figure 3:**
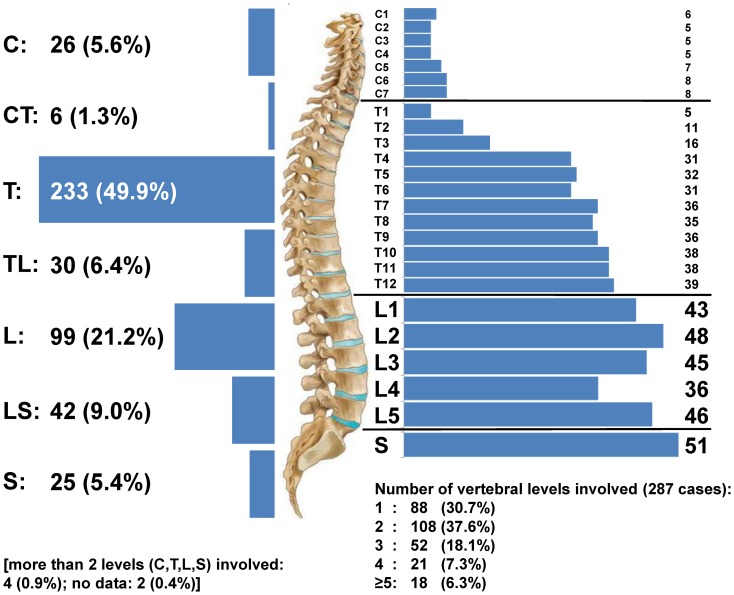
Anatomical allocation of spinal CE. Left: frequency of involved spine levels in 467 cases [C: cervical; CT: cervico-thoracal; T: thoracal; TL: thoraco-lumbal; L: lumbar; LS: lumbo-sacral; S: sacral]. Right: top: frequency of the involved vertebral levels in 303 cases. bottom: number of vertebral levels involved in 287 cases.

Data on the spinal level of the cyst(s) (cervical, cervico-thoracal, thoracal, thoraco-lumbar, lumbar, lumbo-sacral, sacral) was available for 465 of the 467 cases. In 303 of these cases, specific data on the involved vertebral level(s) was available and in 287 of these cases, it was possible to determine the exact number of involved vertebral levels (the discrepancy of these figures is due to the fact that not in all cases with sacral involvement the exact number of involved sacral vertebral levels was reported). The frequency and distribution of the spinal level(s) and individual vertebral level(s) involved is shown in figure 3.

**Figure 4:**
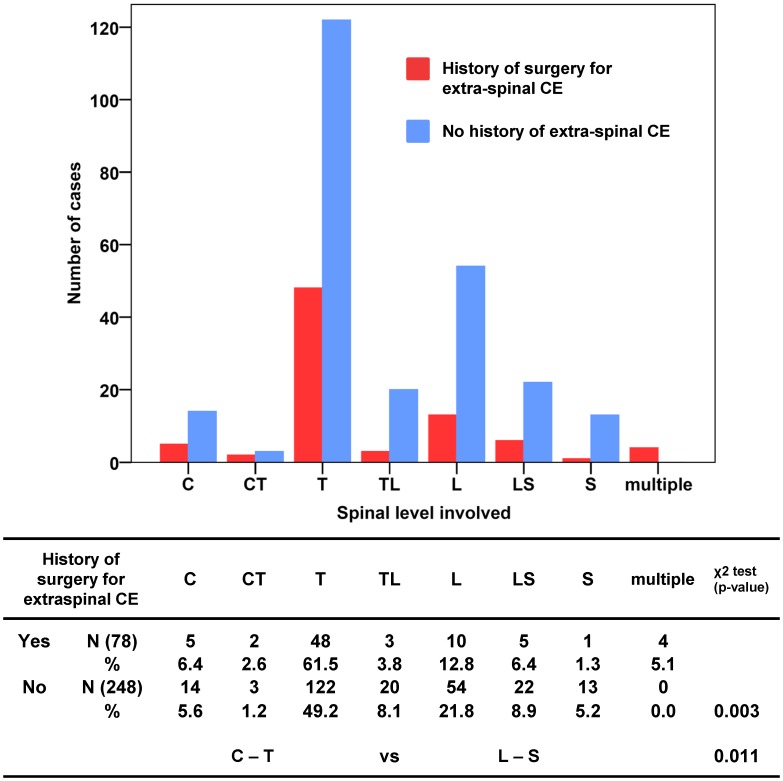
Spinal levels involved in patients with (78 cases) and without (248 cases) history of surgery for extraspinal CE (data on 326 cases).

A subgroup analysis was performed concerning the possible difference in cyst location in cases with a history of extraspinal CE surgery (table 1). Spinal CE cases having a history of previous extraspinal CE surgery were principally operated on for pulmonary CE (table 2) and showed a statistically significant association with upper (thoracic) spine involvement (figure 4).

**Table 1:** Prevalence of previous surgical interventions/concomitant asymptomatic extraspinal CE (data from 467 cases).

Prevalence (n = 467)	Number of cases (%)	Notes
History of previous surgical intervention for spinal CE	36 (7.7)	Recurrence of disease may manifest as late as 29 years following surgery for spinal CE [25]; Chronic recurrent/persisting spinal CE for up to 34 years has been reported [34]
History of surgical intervention for extraspinal CE	78 (16.7)	Time between surgery of extraspinal CE and spinal CE [data available for 24 cases]: median 10 years (range 0.5–28 years)
Newly diagnosed concomitant asymptomatic extraspinal CE during diagnostic workup of spinal CE	6 (1.3)	1× multiple inactive liver cysts, 1× multiple liver cysts (stage III), 1× liver cysts (unspecified), 1× liver & lung cysts (unspecified), 1× liver & kidney cysts (unspecified), 1× cardiac cyst in left ventricle (after 2 previous operations of spinal CE)

**Table 2:** Location of extraspinal CE.

Location of extraspinal CE (n = 96)	Number of cases	%
Lung	38	40
Liver	28	30
Liver or lung (not specified)	7	7
Soft tissue/skeletal muscles	6	6
Thorax wall/ribs	4	4
Intrathoracic, extrapulmonary	4	4
Kidney	3	3
Intraabdominal, extrahepatic	2	2
Intracerebral	1	1
Heart	1	1
Not specified	2	2

Location of the 96 extraspinal hydatid cysts in the 78 spinal CE cases having a history of surgery for extraspinal CE.

**Figure 5:**
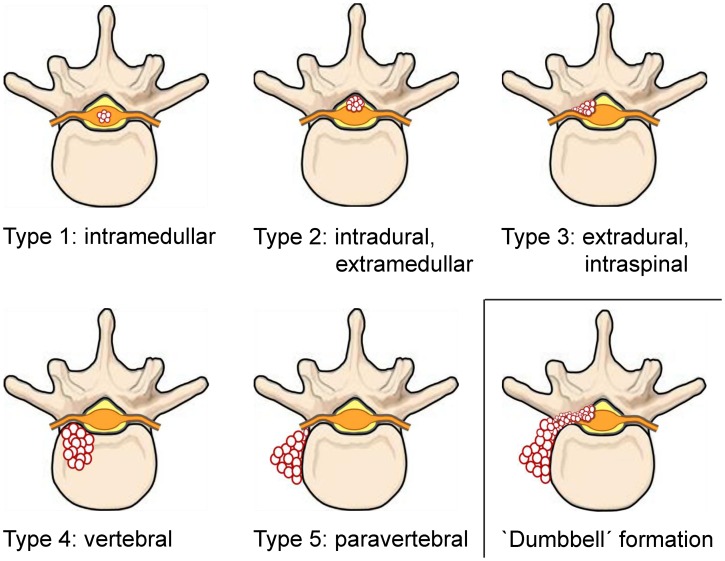
Classification of spinal CE according to the Dew/Braithwaite & Lees classification (type 1–5) and ‘dumbbell’ formation.

**Figure 6:**
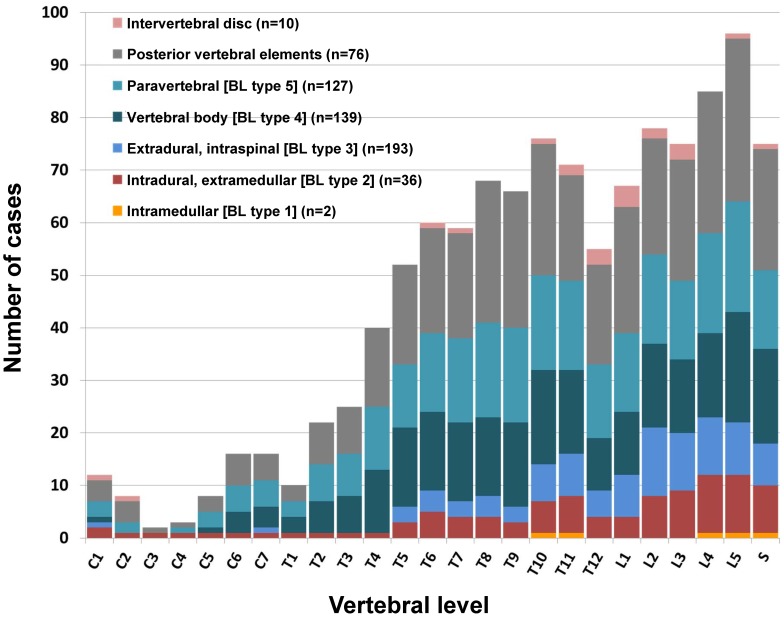
Involved anatomical structures at vertebral level in 230 spinal CE cases.

To evaluate the allocation of spinal CE to the different anatomical structures, we classified the cases according to the Dew/Braithwaite & Lees classification (figure 5) and additionally collected data on the involvement of posterior vertebral elements (pedicles, transverse processes, vertebral arch) and intervertebral disc involvement. Complete data on the involved anatomical structures was available for 230 cases (table 3, 4). Figure 6 shows the involvement of the different anatomical structures at the vertebral level. A frequently reported manifestation of spinal CE is a ‘dumbbell’-formation: a continuous lesion with an intraspinal-extradural and an intrathoracic-paravertebral part, communicating through one or more intervertebral foramina (i.e. a combination of a BL type 3 and a BL type 5 lesion [± additional structures]) (figure 5). As spinal CE presenting with a ‘dumbbell’-formation has frequently been described in the literature, we explored the collected data on the frequency of this manifestation (table 4).

**Table 3:** Anatomical structures involved in 230 spinal CE cases.

Anatomical sites/structures involved in spinal CE (n = 230)	Number of cases	Single cyst	Multiple cysts
	(%)	(%)	(%)
Paravertebral	127	14	104
[BL type 5]	(55.2)	(11.9)	(88.1)
Vertebral body	139	21	104
[BL type 4]	(60.4)	(16.8)	(83.2)
Extradural, intraspinal	193	40	135
[BL type 3]	(83.9)	(22.9)	(77.1)
Intradural, extramedullar	37	14	23
[BL type 2]	(16.1)	(37.8)	(62.2)
Intramedullar	2	1	1
[BL type 1]	(0.9)	(50)	(50)
Posterior vertebral elements	76	7	64
	(33.0)	(9.9)	(90.1)
Intervertebral disc	22	0	10
	(9.6)	(0)	(100)

BL: Braithwaite & Lees classification.

**Table 4:** Number of anatomical sites/structures involved in 230 spinal CE cases.

Number of anatomical sites/structures involved* (n = 230)	Number of cases (%)	‘Dumbbell lesion’ (%)
1	74 (32.2)	-
2	51 (22.2)	20/51 (39.2)
3	53 (23.0)	
4	46 (20.0)	85/105 (81.0)
5	6 (2.6)	
**‘Dumbbell lesion’**	**105 (45.7)**	
without bone involvement	20 (19.0)	
( = 2 structures: extradural-intraspinal & paravertebral)		
with bone involvement	85 (81.0)	
(>2 structures)		

* according to the 7 entities defined in table 4.

**Figure 7:**
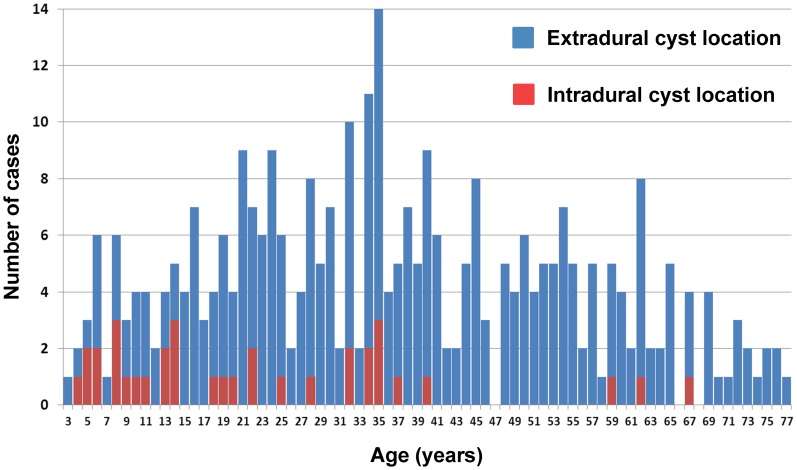
Age distribution of cases with extradural vs. intradural cyst location (data on 325 cases).

We observed a statistically significant difference in the age of patients presenting with intradural (BL type 1 & 2) and extradural cysts (BL type 3, 4 & 5): the median age of patients with extradural cyst location was 36 years (range 3–77 years), while the median age of patients with intradural cysts location was 18.5 years (range 4–67 years) [p<0.0001](figure 7).

## Discussion

### Epidemiology

CE is prevalent throughout most of the world and regional incidence rates of human infection differ widely, depending on the local interaction of man and the natural definitive and intermediate hosts. The greatest prevalence of CE in human and animal hosts is found in countries of the temperate zones, including several regions of Eurasia (the Mediterranean regions, southern and central parts of Russia, central Asia, China), Australia, some parts of America (especially South America) and north and east Africa [8].

Spinal CE is seen in all age groups, with both sexes being affected (figure 2). The median age of 35 years is consistent with published data from larger case series, where the median age was 30 [9], 33 [10], 35 [11], and 36 [12] years respectively. The overall gender distribution of 56.9% male/43.1% female is similar to the distribution found in a large review of 38 Turkish publications covering 111 cases (65.8% male/34.2% female) [13]. However, in our review, case reports and case series originating from very different epidemiological settings were included. Therefore, the analysis may not necessarily apply to specific local situations, where age or gender distributions may differ according to local exposure patterns (with different social, occupational and environmental factors influencing the local interaction pattern of the accidental human intermediate host with the definitive host and the environment).

### Anatomy

#### The route of infection

Animal studies have shown that following oral infection the hatched oncospheres actively penetrate the villi of the jejunal and upper ileal mucosa and it appears that venous as well as lymphatic vessels allow the parasite access to the host's circulatory system [14]. The parasite's predilection for the liver and the lungs is mostly attributed to the filter effect of these organs' capillary beds at ‘first-pass’, but possibly additional host- or parasite specific factors may play a role in the onchosphere's implantation and metacestode development [15].

While extra-visceral CE is generally thought to evolve from arterial dissemination of the oncospheres, some authors have postulated alternative ‘venous routes’ via porto-vertebral shunts and the retrograde passage of the parasite from the inferior vena cava to retroperitoneal- and epidural venous plexuses in spinal CE (e.g. under conditions associated with *Valsalva maneuvers*) [1], [16]–[18].

In large cases series of spinal CE, a predominant involvement of the thoracic (45–50%) and the lumbar spine (20–39%) has been described [13], [19], [20]. Even though the vascular route of infection in spinal CE remains debatable, the predilection for the spine, and especially the thoraco-lumbar region, has been attributed to the dense local vascularisation and the rich blood supply of the vertebral cancellous bone [21].

Upon initial review, our analysis confirms the thoraco-lumbar distribution pattern of spinal CE that is most frequently reported in the literature (figure 3, left). However, the subanalysis alters the picture and shows that the predominant thoracic localisation of spinal CE depends exclusively on the higher number of vertebrae in this segment. The involvement of the individual vertebral levels is rather gradual with an ascending decline (figure 3, right).

Besides the anatomically oriented ‘Dew’-classification (1928 [22]), which is frequently also referred to as ‘Braithwaite & Lees’-classification (1981 [23])(figure 5), spinal CE can be classified according to the route of spinal infection:

1. *Primary haematogenous spinal CE*:
  hematogenous infection of spinal structures at primary infection
2. *Secondary haematogenous spinal CE*:
  hematogenous infection of spinal structures following spontaneous or iatrogenic seeding from extraspinal CE
3. *Secondary ‘per contiguitatem’ spinal CE*:
  direct invasion of spinal structures from extraspinal CE [e.g. mediastinal and paravertebral soft tissue, pleura, lung, ribs, pelvis, posterior paravertebral muscles]
4. *Secondary ‘per continuitatem’ spinal CE*:
  cerebral CE with spontaneous or iatrogenic seeding into the cerebrospinal fluid, leading to intradural spinal seeding

### Primary haematogenous spinal CE

Considering that only 17.9% (120 cases) of all reviewed spinal CE cases had a history of extraspinal CE or were found to have concomitant newly diagnosed extraspinal CE (table 1), it appears that primary spinal CE is more frequent than secondary spinal CE. Even when taking into account that the reviewed case reports often did not mention or provide data on screening investigations for extraspinal CE, and that cases and case series were included, which were published before ultrasound and cross-sectional imaging techniques (CT, MRI) became available, this assumption appears to be justified.

### Secondary haematogenous spinal CE

Whether spinal CE in patients with a history of extraspinal CE results from simultaneous primary infection (spinal and extraspinal infection acquired simultaneously on primary infection), arises from secondary hematogenous seeding of extraspinal CE or constitutes a new exogenous infection is difficult to say.Tapia and colleagues stated that osseous CE is probably acquired in childhood and remains clinically latent even for more than 40 years and typically manifests in adults [24]. Local recurrence of spinal CE has been reported to occur up to 29 years after surgery [25]. Discriminating dormant primary spinal infection from dormant secondary hematogenous seeding to the spine is impossible. The only way to prove exogenous reinfection would demand genotyping of the primary and secondary site of infection (Note: genetic characterization of the parasite was not reported in any of the reviewed spinal CE cases).

When reviewing the collected data, secondary spinal CE arising from spontaneous haematogenous seeding of extraspinal CE might exist, but is probably very rare: we found only 6 cases of spinal CE where concomitant asymptomatic extra-spinal CE was reported (table 1). In 5 of these cases, spinal seeding from visceral CE may have occurred (in 2 cases the cyst stage supports the assumption that visceral CE anteceded spinal CE). The 6^th^ case, an intraventricular cardiac cyst diagnosed after 2 previous surgical interventions for spinal CE, was the only case we found indicating haematogenous seeding following surgery of spinal CE.

Haematogenous seeding following surgery of extra-spinal CE has been reported and is generally considered to be the most common route in spinal infection [26]. We found a history of previous surgical intervention(s) for extraspinal CE in 16.7% of the spinal CE cases (table 1). This figure is comparable to the extraspinal CE prevalence of 14.4% reported in a Turkish series of 111 spinal CE cases [13].

Bearing in mind that hepatic CE is typically more common than pulmonary CE (see introduction), it is interesting that most cases of spinal CE with a history of surgery for extraspinal CE were operated on for pulmonary CE (table 2). Even though the available data is limited and does not permit deeper analysis, three possible explanations could be discussed: 1. the risk for spinal seeding following surgery of pulmonary CE might be higher than in surgery of hydatid cysts at other locations; 2. pulmonary CE might be an indicator for a porto-systemic route of primary infection rather than being causally related; 3. pulmonary CE might be an indicator for an inhalative route of primary infection: in 1965 Borrie and colleagues demonstrated that inhalation of *E. granulosus* eggs can lead to pulmonary hydatid disease in sheep [27]. Therefore, even though never proven for the human host, inhaled eggs could theoretically enter the pulmonary circulation, disseminate systemically and reach the spine.

The performed subgroup analysis of the vertebral level involvement in cases with and without a history of surgery for extraspinal CE show a statistically significant difference: previous surgery for extraspinal CE appear to be more frequently associated with thoracic vertebral involvement (figure 4). This observation would indirectly support the speculation that primary hematogenous spinal infection of especially the lower parts of the spine occurs via porto-vertebral shunts (see above).

Irrespective of the route of infection the putative ‘dormant’ period of spinal CE appears to be very long (table 1) and emphasizes long-term follow-up. However, the available data does not support standard screening of patients with extraspinal CE for concomitant asymptomatic spinal CE.

### Secondary ‘per contiguitatem’ spinal CE

In most cases the exact primary implantation site of the parasite and the primary affected spinal structure remains unclear and the disease is only diagnosed after several anatomical structures become affected (table 4). The summarized data (table 4; figure 6) suggests that the parasite's primary implantation site can be either the vertebral bone (with secondary extra-osseous spread to the paravertebral and intraspinal space) or the paravertebral or intraspinal soft-tissue (with secondary infiltration of the vertebral bone). While all spinal structures can be infiltrated in the course of disease, no case has been published reporting dura infiltration or penetration.

### Secondary ‘per continuitatem’ spinal CE

Secondary ‘per continuitatem’ spinal CE appears to be very rare: we found 2 case reports of spinal seeding following surgery of cerebral CE, but no case report of spontaneous spinal seeding from cerebral CE [13], [28].

#### Local evolution of spinal CE

Depending on the primarily infected anatomic structure the evolution of spinal CE differs.

In CE arising from vertebral bone, growth of the parasite is generally slow (due to the resistant nature of bone) and characterized by aggressive bone infiltration. Unlike in extraosseous CE, pericyst formation does not occur in osseous CE and the resulting microvesicular polycystic infiltration of the bone follows the line of least resistance along the intratrabecular spaces of the vertebra [5], [16], [23], [29]–[31]. Next to the direct local pressure erosion of bone, pressure on blood vessels within the bone (causing local ischemic necrosis) contributes to bone destruction [16]. Destructive growth eventually leads to symptomatic disease when the cysts breach the vertebral cortex and infiltrate neighbouring structures (like the spinal channel) or spontaneous fracture of the vertebra occurs. Once the cysts extend anteriorly, laterally or posteriorly beyond the vertebral body they show eccentric spherical growth as they do in soft tissues [30].

In CE arising from the spinal- or paraspinal soft tissues, the growth pattern is primarily eccentrically spherical and follows the line of least resistance. Secondary bone erosion or infiltration is not uncommon, but generally growth follows along the spinal channel, the intervertebral foramina, and the vertebral column. This growth pattern eventually leads to the formation of a ‘dumbbell’-lesion (figure 5), which was observed in 45.7% of the reviewed cases (table 4).

Intervertebral disc involvement in spinal CE is generally rare and the discs usually remain unaffected as the cysts tend to propagate beneath the periosteum and the ligaments [16], [30]. Disc involvement is considered to be a late feature following extensive vertebral destruction in prolonged disease. Among the reviewed cases, only 22 (9.6%) were reported to show disc involvement (table 3). Of note is that 12 of these cases were reported from a single study involving 13 patients who underwent a total of 42 major surgical procedures, indicating advanced stages of disease [10]. One case report has been published on paravertebral CE infiltrating two contiguous intervertebral disks without bone involvement [32].

Growth of intradural-extramedullar cysts is eccentric and follows the line of least resistance along the dural sack. Compared to extradural CE, intradural CE is more frequently limited to a single cyst (table 3) and infection appears to present at a younger age (figure 7), which is most likely explained by the earlier appearance of neurological symptoms due to cord compression. Interestingly, 2 of the 3 oldest patients presenting with intradural CE (59 and 67 years old; figure 7) had a history of previous surgery for extraspinal CE, which (in addition to the finding of multiple intradural cysts) strongly suggests secondary hematogenous seeding rather than primary infection [33], [34].

Intramedullar CE is very rare and (besides a reference to a disputable case reported by Montansey in 1827 [35]) we found only two published cases [36], [37]. In one of the two cases intradural-extramedullar and intramedullary cysts were concomitantly present [37].

While cysts might adhere to the dural sack, infiltration or penetration has not been reported in any case of intra- or extradural CE. We found two published cases of primary concomitant extradural and intradural-extramedullar CE [38], [39]. Secondary intradural seeding following surgery of extradural CE has been reported [33], [34].

#### Differential diagnosis

The course of symptomatic disease might range from acute onset to prolonged clinical courses where the diagnosis is often only made many years or even decades after the first appearance of symptoms [40]–[42]. Lacking characteristic signs and symptoms, spinal CE may manifest with any symptom linked to vertebral bone destruction or spinal cord compression, but a long history of back pain and/or subacute symptoms related to spinal cord or spinal nerve compression (radicular pain, peripheral sensitivity loss, sphincter disturbance, bladder dysfunction, paraparesis, paraplegia) are the most frequent [1], [9],[13],[19],[43],[44].

Depending on the primarily involved anatomical structure, the differential diagnosis of spinal CE is diverse: tuberculosis (Pott's disease), pyogenic infection (osteomyelitis), brucellosis, fibrous dysplasia, simple or aneurysmal bone cysts, malignancy (e.g. multiple myeloma, chondrosarcoma) or spinal metastasis. Various tumors may present with a dumbbell formation (e.g. chondrosarcoma, neurilemmoma, neuroblastoma [45], [46]). The differential diagnosis of intraspinal cystic lesions includes dorsal arachnoid diverticula and meningoceles and the differential diagnosis of intradural cystic lesions includes arachnoid cysts, syringomyelia and neurocysticercosis. Intradural-extramedullar or intramedullar cysticerci may mimic CE and have been described even in the absence of concomitant parenchymal brain lesions [47]–[49]. Besides the rare cases of spinal cysticercosis some other, even rarer, cestode infections may involve spinal structures: cases of spinal alveolar echinococcosis (*E. multilocularis*) [50]–[57] and spinal sparganosis (*Spirometra species*) [58], [59] have been published. Cases of spinal coenurosis (*Taenia multiceps*, *T. crassiceps*, *T. serialis*) or spinal South American neotropical echinococcosis (*E. oligarthrus*, *E. vogeli*) are possible, but to our knowledge no such cases have been published.

In particular, the intra-operative finding of pus-like fluid in advanced vertebral CE, termed ‘ossifluent abscess’, can lead to the misdiagnosis of vertebral tuberculosis or pyogenic infection [60]–[64].

Within endemic regions, spinal CE is an important differential diagnosis in spinal cord compression syndrome: CE was reported to be responsible for 3.8% (Turkey), 4.5% (Marocco) and 14% (Tunisia) of all cases presenting with cord compression syndrome [65], [66].

### Conclusion

Despite significant advances in diagnosis and treatment of CE many aspects, including the parasite's predilection for the spine in osseous CE, remain poorly understood.

Spinal CE primarily affects the thoraco-lumbar spine, involving the individual vertebral levels with gradually ascending decline.

Contrary to common perception, primary spinal CE appears to be more frequent than secondary spinal CE.

It appears that the affected vertebral level in spinal CE differs in patients with and without history of surgery for extraspinal CE. Previous surgery for extraspinal CE appears to be more frequently associated with thoracic vertebral involvement.

Patients with intradural CE present at a younger age than patients with extradural CE.

Possibly future studies will be able to identify parasite and/or host specific parameters to provide molecular genetic based explanations for the interindiviudal differences in local manifestation and evolution of CE.

## Supporting Information

Checklist S1 PRISMA Checklist. 27-item checklist for systematic reviews.
(DOC)

Flowchart S1 PRISMA Flow Diagram. Flow of information through the different phases of the systematic review.
(DOC)

References S1 Reference list of included and excluded publications.
(ZIP)
